# Variation of rhizosphere microbial community in continuous mono-maize seed production

**DOI:** 10.1038/s41598-021-81228-1

**Published:** 2021-01-15

**Authors:** Yunchen Zhao, Wenjiang Fu, Changwei Hu, Guangquan Chen, Zhanwen Xiao, Yuru Chen, Zhijiang Wang, Hongyu Cheng

**Affiliations:** 1 School of Agriculture and Ecological Engineering, Hexi University, Zhangye, 734000 Gansu China; 2 College of Biological, Chemical Science and Engineering, Jiaxing University, Jiaxing, 314000 Zhejiang China; 3 School of Food Science and Pharmaceutical Engineering, Nanjing Normal University, Nanjing, 210000 Jiangsu China

**Keywords:** Microbial communities, Pathogens

## Abstract

Soil microbe is crucial to a healthy soil, therefore its diversities and abundances under different conditions are still need fully understand.The aims of the study were to characterize the community structure and diversity of microbe in the rhizosphere soil after continuous maize seed production, and the relationship between the disease incidence of four diseases and the variation of the rhizosphere microbe. The results showed that different fungal and bacterial species were predominant in different cropping year, and long-term maize seed production had a huge impact on structure and diversity of soil microbial. Ascomycota and Mortierellomycota were the dominant fungal phyla and *Mortierella* and *Ascomycetes* represented for a large proportion of genus. A relative increase of *Fusarium* and *Gibberella* and a relative decrease of *Mortierella, Chrysosporium, Podospora,* and *Chaetomium* were observed with the increase of cropping year. Pathogenic *Fusarium, Curvularia, Curvularia-lunata, Cladosporium, Gibberella-baccata*, and *Plectosphaerellaceae* were over-presented and varied at different continuous cropping year, led to different maize disease incidence. Proteobacteria and Actinobacteria ranked in the top two of all bacterial phyla, and genus *Pseudarthrobacter, Roseiflexus* and *RB41* dominated top 3. *Haliangium* and *Streptomyces* decreased with the continuous cropping year and mono-cropping of maize seed production increased disease incidence with the increase of cropping year, while the major disease was different. Continuous cropping of maize seed production induced the decrease of protective microbe and biocontrol genera, while pathogenic pathogen increased, and maize are in danger of pathogen invasion. Field management show great effects on soil microbial community.

## Introduction

Continuous mono maize seed cropping is very prevalent in Northwest of China for its high interest and unique geographical advantage, some fields even lasting more than 30 years, and resulted in serious obstacle of the crop^1^. Maize crop is infected by approximately 65 pathogens including fungi, bacteria, and viruses^2^. In the continuous mono maize seed cropping system, the maize is generally suffered with high incidence of the disease like root rot, seedling blight, stem rot, and ear rot and as a consequence the yields decreased 6.75–9.36%^3^. It’s well known that those diseases are caused by numerous pathogens, such as *Fusarium*, *Rhizoctonia solani* and *Pythium* etc., resulted in large economic losses^4^, limiting quality improvement^5^, and restrained the development of industry chain and farmers’ planting enthusiasm.

Soil microorganisms are key drivers of plant productivity in terrestrial ecosystem^6^. Studies showed that rhizosphere fungi play an essential role in plant growth and health of the plant-soil ecosystem^7^. Soil microbial community is thought to be responsible for biological processes that are necessary for maintaining a healthy soil and suppressing plant diseases^8,9^. Furthermore, it has been shown that a decrease in soil microbial diversity was responsible for the development of soil-borne plant disease^10,11^ which addressed that the differences in the rhizosphere microbial community may contribute to the differences in resistance to many disease. Generally, soil suppression relates to the biomass, activity and diversity of microbe at the community level and was reported to negatively correlate with invader survival^12,13^.

However, phyto-pathogenesis was complex, different plant species, location, and microbe community may lead to different disease incidence and resistance. Studies showed that pathogenic bacteria of maize rot of different part involved more than 30 species of *Fusarium*^14^*, Penicillium, Aspergillus,* and *Rhizopus Nigricans* etc. *Fusarium* and *Aspergillus* infection caused root rot at first, then stem rot, then ear rot at the next coming years under some location and conditions^15^. To date, the effective methods to effectively control those diseases are still scarce. In addition, there is lack of knowledge on microbial community structure and diversity of rhzisophere of continuous maize seed production. Hence, analysis of the community composition and structure of rhizosphere microbe of maize seed continuous cropping fields, and identify the predominant pathogens causing maize disease of high incidence during a season are helpful to prevent those disease. Therefore, considerable interest should be paid on monitoring the composition and variation of soil microbial communities in different continuous mono cropping year of maize seed production.

In present study, variation of rhizosphere microbial community in different continuous cropping year of maize seed production was investigated to characterize fungal and bacterial communities in the rhizosphere soils. We aimed to (1) evaluate the impact of mono-cropping on diversity and structure of soil microbial communities in the continuous maize seed production, (2) determine the dynamic change of pathogenic bacteria of different cropping year, (3) estimate whether the continuous mono-maize seed production causing serious pathogenic bacteria accumulation in different mono-cropping year, and (4) reveal the relationship between root rot, stem rot, seedling blight, ear rot and the dynamic change of microbe community of maize seed production system.

## Results

### The disease incidence during growth stage and the severity

Generally, the disease incidence increased with the increase of continuous cropping year with exception of 30 years with stubble (Table 1). The highest disease incidence of seedling blight and ear rot was 8.23% of 20 years continuous cropping and 12.92% of 30 years continuous cropping without field maize, respectively. The ear rot incidence of continuous cropping for 5 years was lower than the others, and no difference was observed between 5 and 10 years cropping. The highest disease incidence of root rot and stem rot was 20 year continuous cropping of 9.58% and 13.55%, respectively. Obviously, continuous mono-cropping of maize seed production could increase disease incidence with the increase of cropping year, while the incidence of four diseases differed among different continuous cropping year (Table 1).

**Table 1:** The disease incidence (%) of different continuous cropping year.

Disease	A	B	C	D	E	F	G
Seedling blight	4.08 ± 0.09d	3.29 ± 0.26e	7.83 ± 0.93a	8.23 ± 0.65a	5.18 ± 0.53c	6.25 ± 0.57b	2.95 ± 0.23e
Root rot	2.33 ± 0.22e	3.44 ± 0.31d	4.57 ± 0.35d	9.58 ± 0.94a	8.15 ± 0.72b	8.21 ± 0.68b	7.23 ± 0.14c
Stem rot	6.82 ± 0.55d	10.11 ± 0.08b	8.68 ± 0.67c	13.55 ± 0.14a	12.03 ± 0.09a	10.11 ± 0.11b	9.25 ± 0.07bc
Ear rot	3.33 ± 0.43d	4.18 ± 0.46d	5.22 ± 0.38c	7.24 ± 1.1b	8.64 ± 0.71b	12.92 ± 1.1a	12.02 ± 0.28a

All the number were expressed with standard error, lowercase letter in the same line means significant difference at *p* < 0.05.

Most of the disease severity of different plots of different cropping year was characterized of level 1 or 2. It can be seen that the disease severity of seedling blight, stem rot and ear rot, which was the common disease in the area of maize seed production, were high in most cropping years. The disease severity of ear rot increased with the cropping year, while the disease severity decreased at 30 years with field maize of seedling blight and root rot. The disease severity of root rot was relative slight than that of others disease in almost all different cropping years (Table 2).

**Table 2:** The severity of different disease in different continuous cropping year.

Disease severity	A	B	C	D	E	F	G
Seedling blight	2	2	2	2	2	2	1
Root rot	1	1	1	2	1	2	1
Stem rot	2	1	2	2	1	2	1
Ear rot	2	1	2	2	2	2	2

1 means level one, 2 means level two, 0 means no disease incidence.

### The community diversity of fungi after mono maize seed cropping

**Figure 1:**
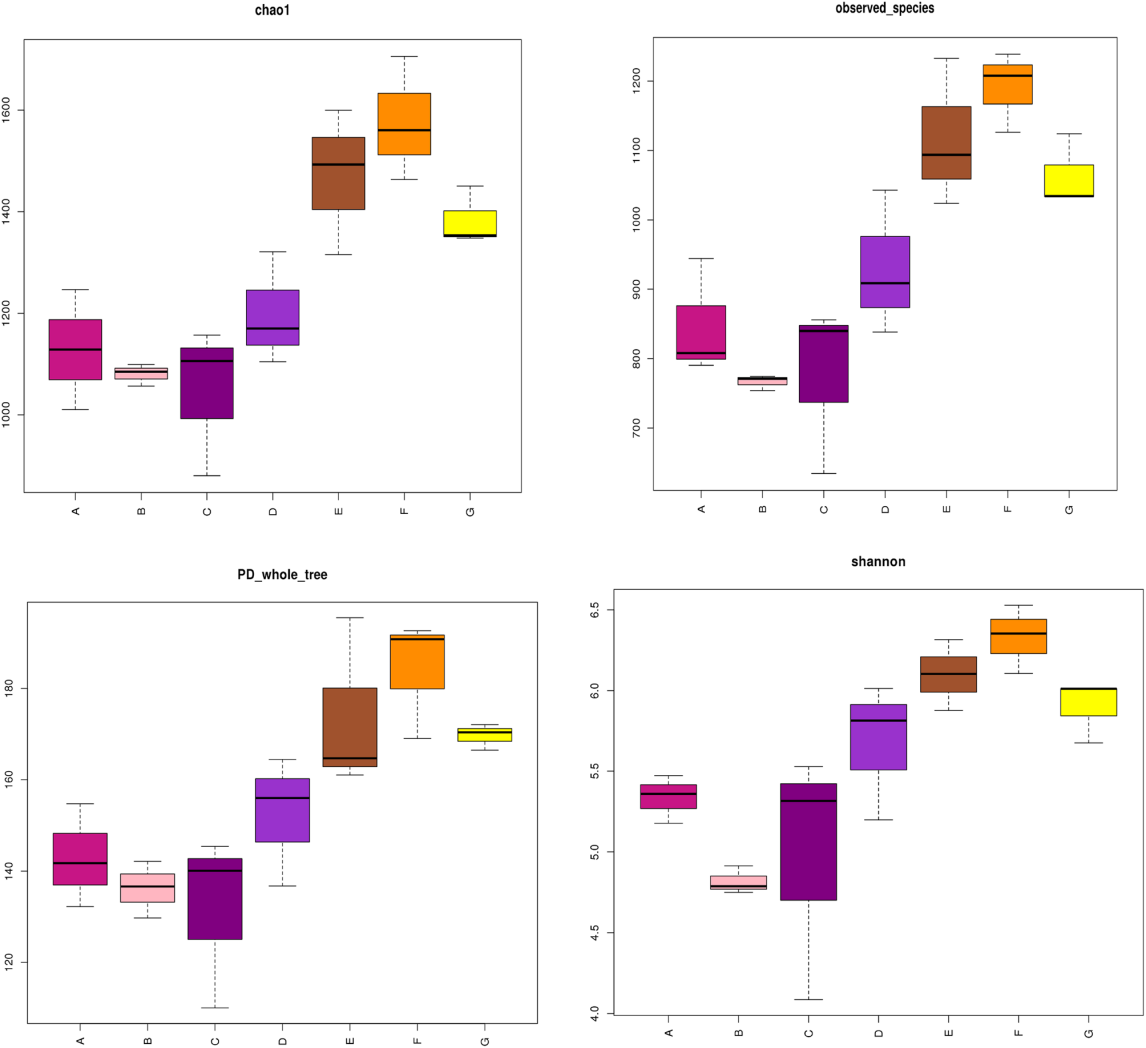
Chao1, observed-species, PD_whole_tree, and Shannon index of fungi of different continuous cropping year. Uppercase of A, B, C, D, E, F and G represent 5,10,15, 20, 25, 30 years, and 30 years with stubble cropping, respectively.

After sequencing DNA isolated from the samples by using high-throughput DNA sequencing, 118, 000 optimized sequences with average lengths of 200–260 bp were retained through filtered from 120,000 valid ITS sequences. The Chao 1, the observed-species, the PD-whole-tree, and Shannon index of the samples were chosen to represent the real diversity of the microbes in the samples (Fig. 1).

**Figure 2:**
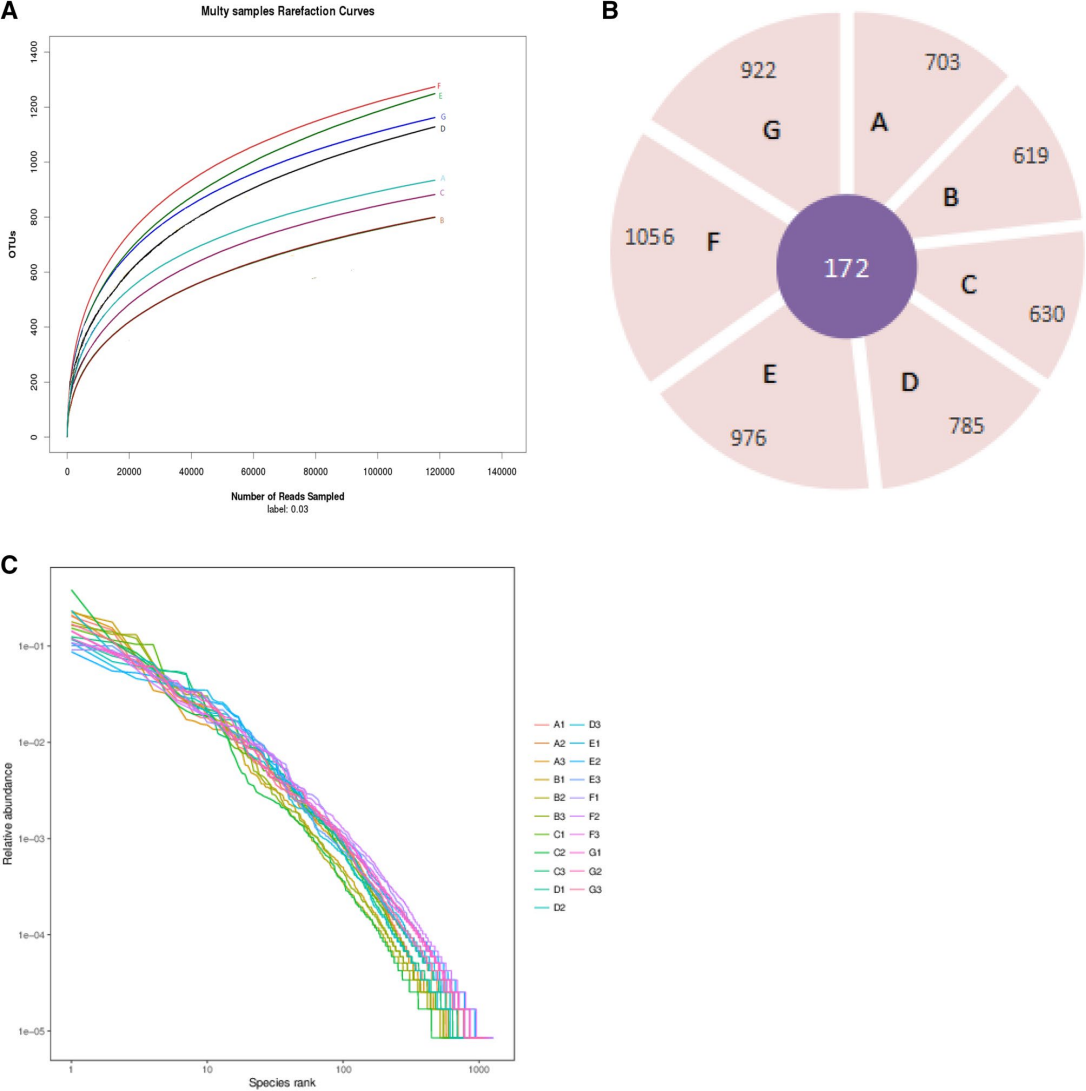
(**A**) Rarefaction curves of OTUs clustered at < 97% sequence identity for 21 different rhizoshpere soil samples of corn seed production from 5, 10, 15, 20, 25, 30 and 30 years of continuous cropping. Uppercase of A, B, C, D, E, F and G represent 5, 10, 15, 20, 25, 30, and 30 years, respectively. The subscript below the letter represent three duplicates of the same cropping year. The OTUs reads of different samples are shown in different colors. (**B**) Flower graph of OTUs of different continuous cropping year. Uppercase A, B, C, D, E, F, and G represent 5, 10, 15, 20, 25, 30, 30 years, respectively. The core was the common OTUs for different treatment. (**C**) OTUs relative abundance curves for the OTUs in the 21 different corn seed production rhizoshpere soil samples from 5, 10,15, 20, 25, 30 and 30 (with field corn) years of continuous cropping. Uppercase A, B, C, D, E, F, and G represent 5, 10, 15, 20, 25, 30, and 30 years, respectively. The subscript below the letter represent three duplicates of the same cropping year. The OTUs reads of different samples are shown in different colors.

The Chao1, PD-whole tree, Shannon index was indices at 97% similarity. The highest and the lowest Shannon index, observed-species, Chao1 were observed in 30 years and 10 years cropping soil, respectively (Fig. 1). The number of OTUs observed increased with the number of sequence sampled, and reached the near plateau region at 97% similarity level.The rarefaction curves trended to be increased continuously as the number of sequences increased (Fig. 2A), and with exception of 172 common OTUs, the upper limit of OTUs for 5, 10, 15, 20, 25, and 30 cropping year were 703, 619, 630, 785, 976, 1056 and 922, respectively (Fig. 2B). The rank-abundance curves are all decreased and flatted at the end, and ranged higher than 10 yeas on the x-axis, showing a higher abundance in 30 and 25 years, with the highest for 30 years (Fig. 2C). Continuous cropping enriched the fungal community diversity and richness, and the diversity increased over continuous cropping years with exception of 10 years cropping.

### The relative abundance of fungi community

**Figure 3:**
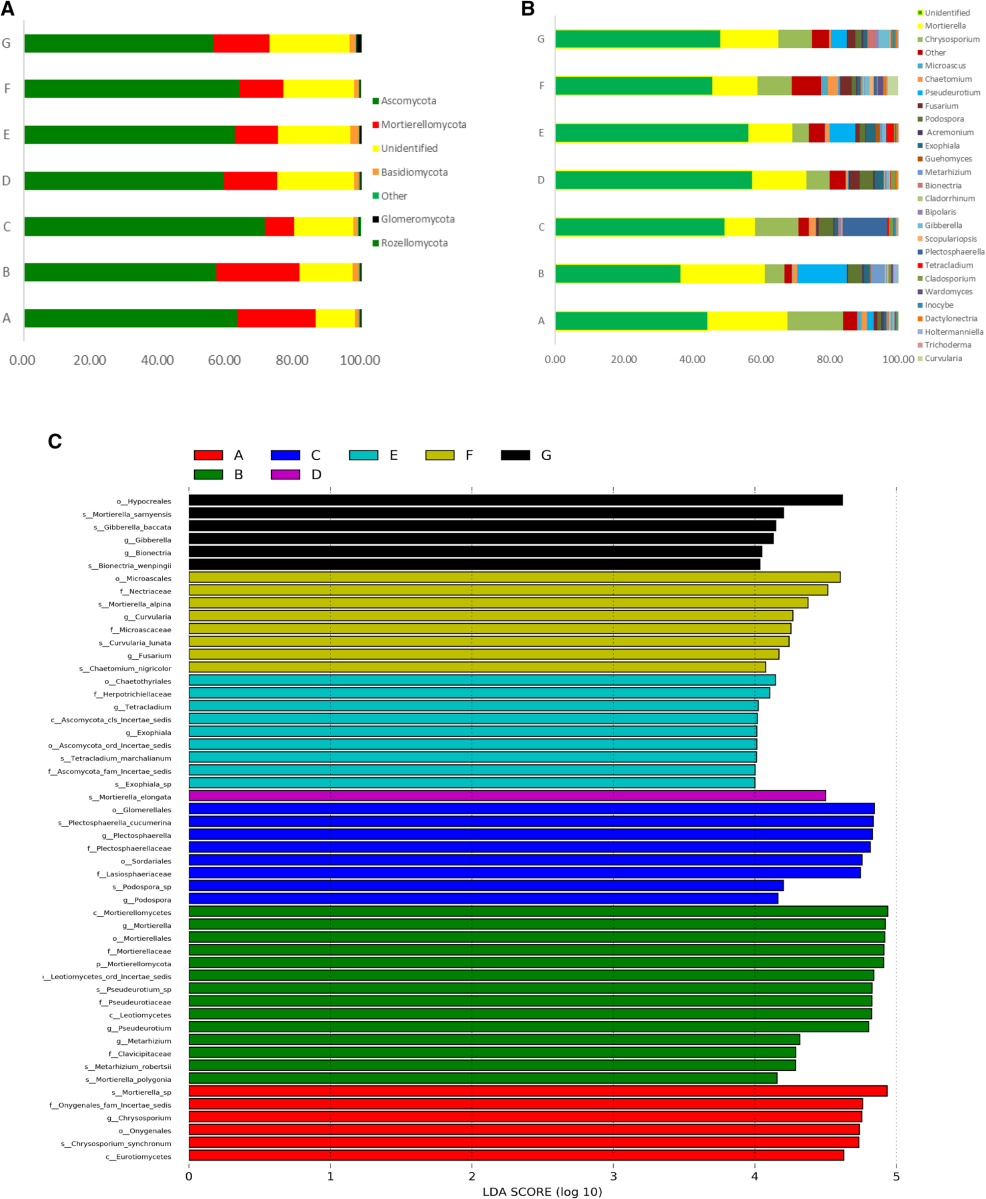
(**A**) Composition of the different fungal communities at the phylum level in seven rhizosphere soil samples. The relative abundance of different fungal phylum within the different communities are shown in different colors. Unidentified means there was no taxonomic information, and Other means many function group. (**B**) Composition of the different fungal communities at the genus level in seven rhizosphere soil samples. The relative abundance of different fungal genus within the different communities are shown in different colors. Unidentified means there was no taxonomic information, and Other means many function group. (**C**) LDA score of the fungi of different cropping year. The figure shows species whose LDA Score is greater than the set value (LDA score > 2, less-strict set to 2; More-strict was set as 4), that is, biomarkers with statistical differences between groups. The length of the bar chart represents the significance of the different species.

The diversity and relative abundance of the rhizosphere fungal community was significantly different with different continuous cropping year. At phylum level, most fungal sequences were dominated distributed at Ascomycota, Mortierellomycota, Basidiomycota, Glomeromycota, and Rozellomycota. Especially, Ascomycota was the most dominant fungal phylum and represented 65.98% on an average of all fungal DNA sequence. Five known fungal phyla accounted for 76.09–89.01% on an average of all. There still presented 12.45–23.91% unidentified and others fungal phyla of all, and unidentified phylum increased with the increase of continuous cropping year (Fig. 3A). At the genus level, the abundance of the sorting results, ordered from high to low, were *Mortierella, Chrysosporium, Pseudeurotium, Fusarium, Podospora, Chaetomium, Exophiala, Bipolaris, Gibberella, Acremonium, Clasosporium, Microascus, Cladorrhinum, Guehomyces, Scopulariopsis, Plectosphaerella, Wardomyces, Tetracladium, Inocybe, Holtermanniella,* and *Bionectria* that belongs to different phylum. Considerable fungi genus at rhizosphere soil were unclassified and showed a relative increase in 20 years cropping. A certain proportion of potential pathogenic pathogen of *Fusarium, Gibberella, Cladosporium, Acremonium,* and *Bipolaris* was detected in the rhizosphere soil of different continuous cropping year. It is worthy note that the relative abundance of *Fusarium, Gibberella, Cladosporium, Exophiala* and the relative depletion of *Mortierella, Podospora, Chrysosporium* and *Chaetomium* at different continuous cropping year (Fig. 3B). According to Lefse analysis, speicies *Gibberella-Baccata* was enriched in the rizosphere soil of 30 years cropping with 3 years field maize planting history. The key genus as *Fusarium, Curvularia, Currlaria-lunata* were dominated in 30 years continuous cropping, and genus *Chaetochyriales* was dominated in 25 years. Species *Mortierella-Elongata* in the soil of 20 years cropping, and genus *plectosphaerellaceae* in the soil of 15 years continuous cropping were more enriched than the other treatments. Class Mortierellamycetes and species Mortierella_sp were over-represented in the rhizosphere soil of 10 and 5 years continuous cropping year, and these key phyla potentially contributed to the structural segregation of different years (Fig. 3C).

### The community diversity of bacteria after mono continuous cropping

**Figure 4:**
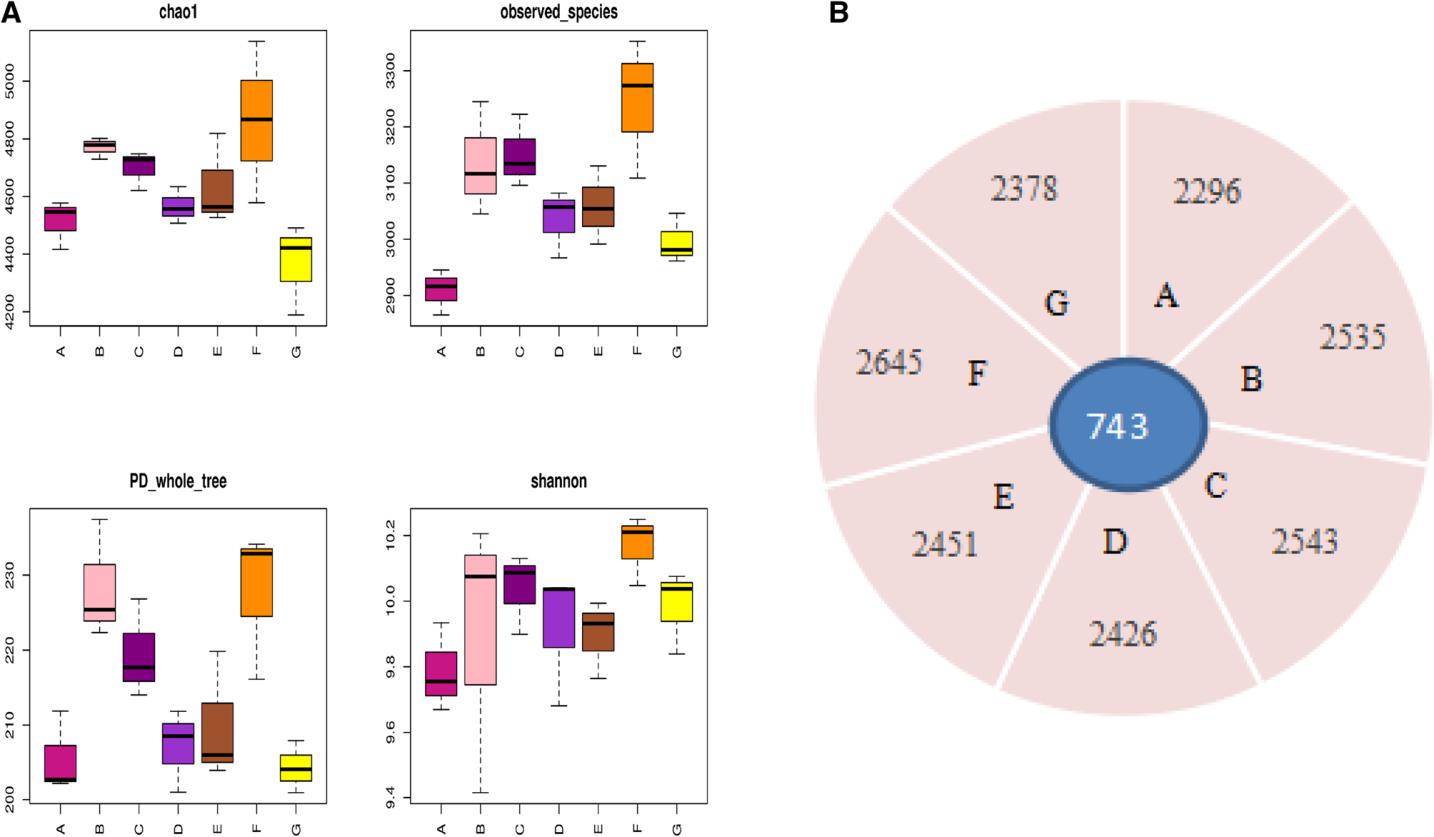
(**A**) Chao1, observed-species, PD_ whole _tree, and Shannon index of bacteria of different continuous cropping year. Uppercase of A, B, C, D, E, F, and G represent 5, 10, 15, 20, 25, 30, and 30 years cropping, respectively. (**B**) Venn diagram of the bacteria of different continuous cropping year. A, B, C, D, E, F, and G represents 5, 10, 15, 20, 25, 30, and 30 years cropping, respectively.

The results reflected significant differences among the samples in bacteria community composition (*P* < 0.05), and a higher Chao1and Shannon index over 30 years and 10 years cropping with the highest for 30 years cropping without field maize were observed. Twenty-five years cropping showed the highest, while 5 years cropping showed the lowest observed-species and PD tree (Fig. 4A). Eighteen thousand optimized sequences with average lengths of 200–260 bp were retained through filtered from 21, 000 valid sequences. The rarefaction curves trended to be decreased as the number of sequences increased, and the upper limit of OTUs 0.03 were 2735, 2952, 2415, 2655, 2679, 3220 and 2805 respectively. The Venn diagram showed that there were 743 common OTUs among the continuously cropped soil samples and 2296, 2535,2543, 2426, 2451, 2645 and 2378 else OTUs were found in 5, 10, 15, 20, 25, 30, and 30 years continuous cropping (Fig. 4B). As revealed by high throughput sequencing that continuous cropping induced different number of OTUs and the bacterial diversity and structure of community varied among different continuous cropping years.

### The relative abundance of bacteria of rhizosphere soil

**Figure 5:**
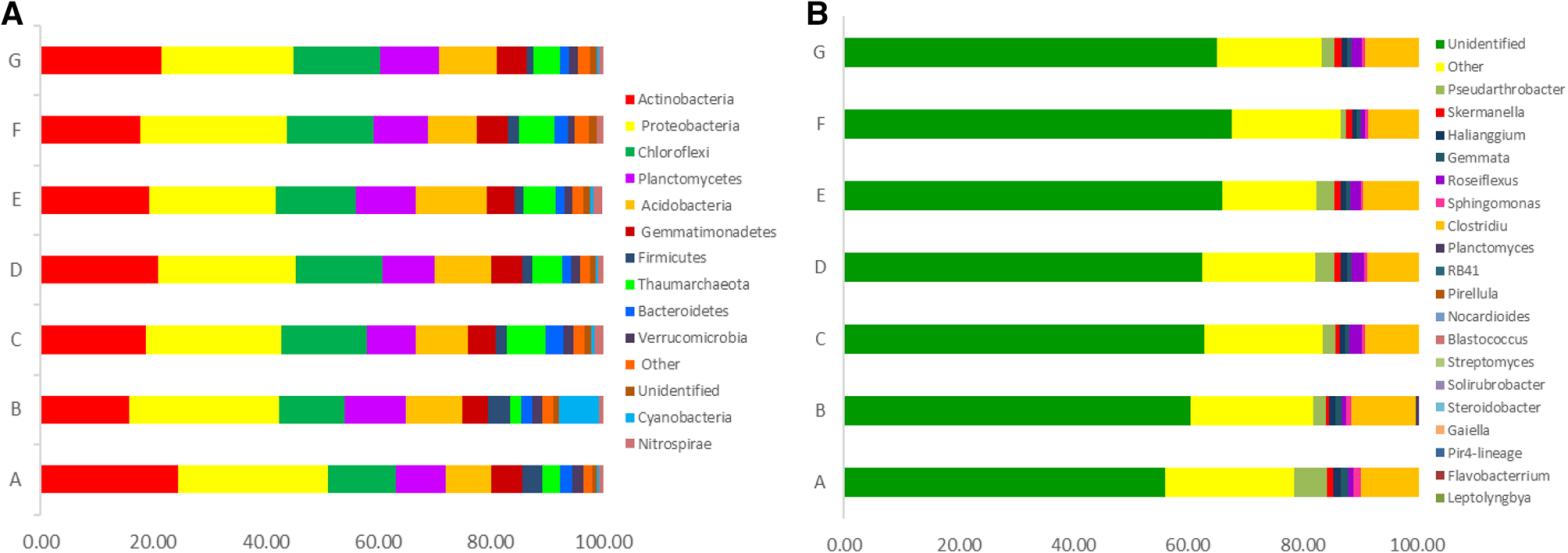

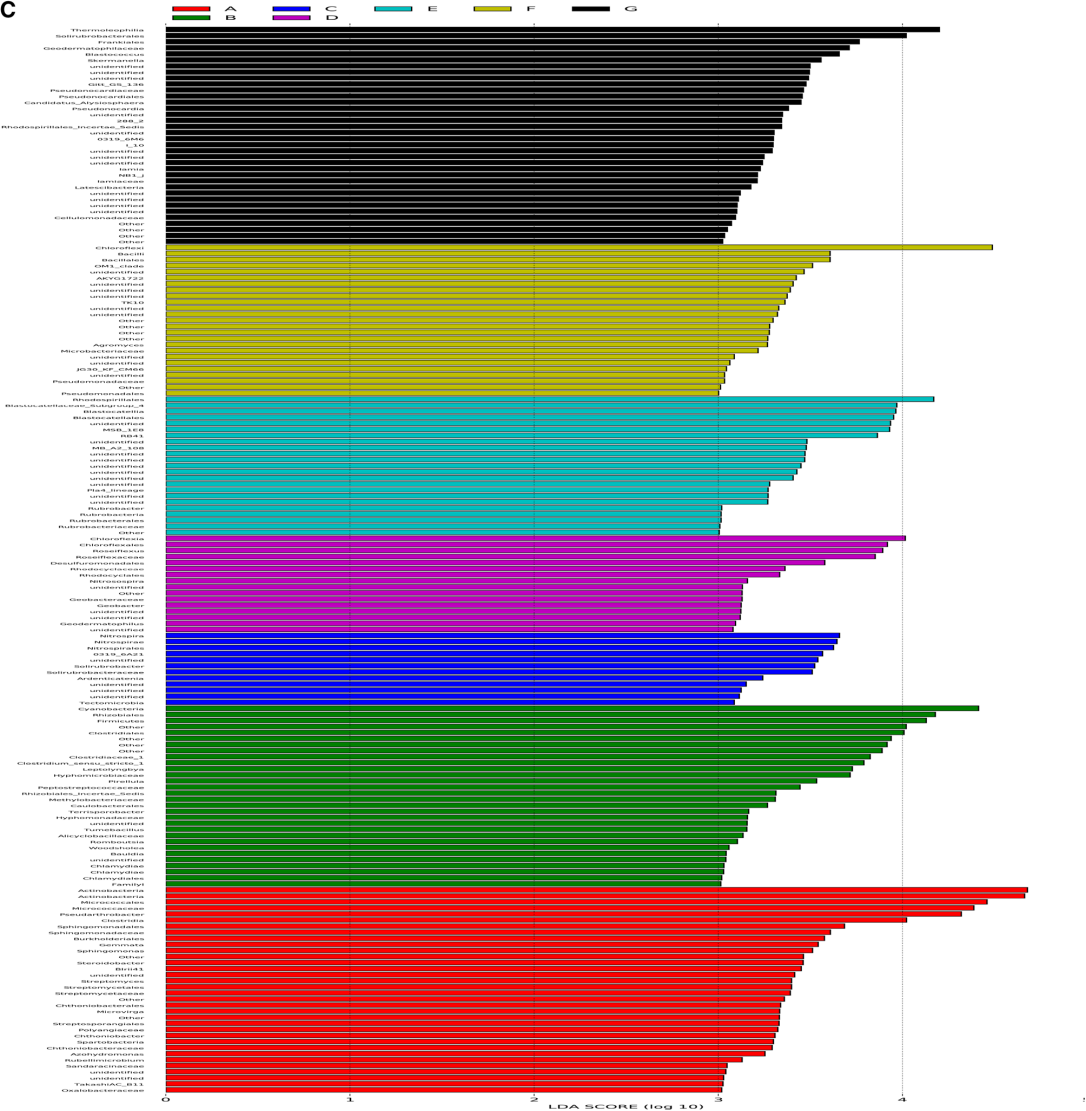
(**A**) Bacterial Phylum of different cropping years based on mean read numbers per sample. (**B**) Bacterial genus of different cropping years based on mean read numbers per sample. (**C**) LDA score of the bacteria of different cropping year. The figure shows species whose LDA Score is greater than the set value ( LDA score > 2, less-strict set to 2; More-strict was set as 4), that is, biomarkers with statistical differences between groups. The length of the bar chart represents the significance of the different species.

**Figure 6:**
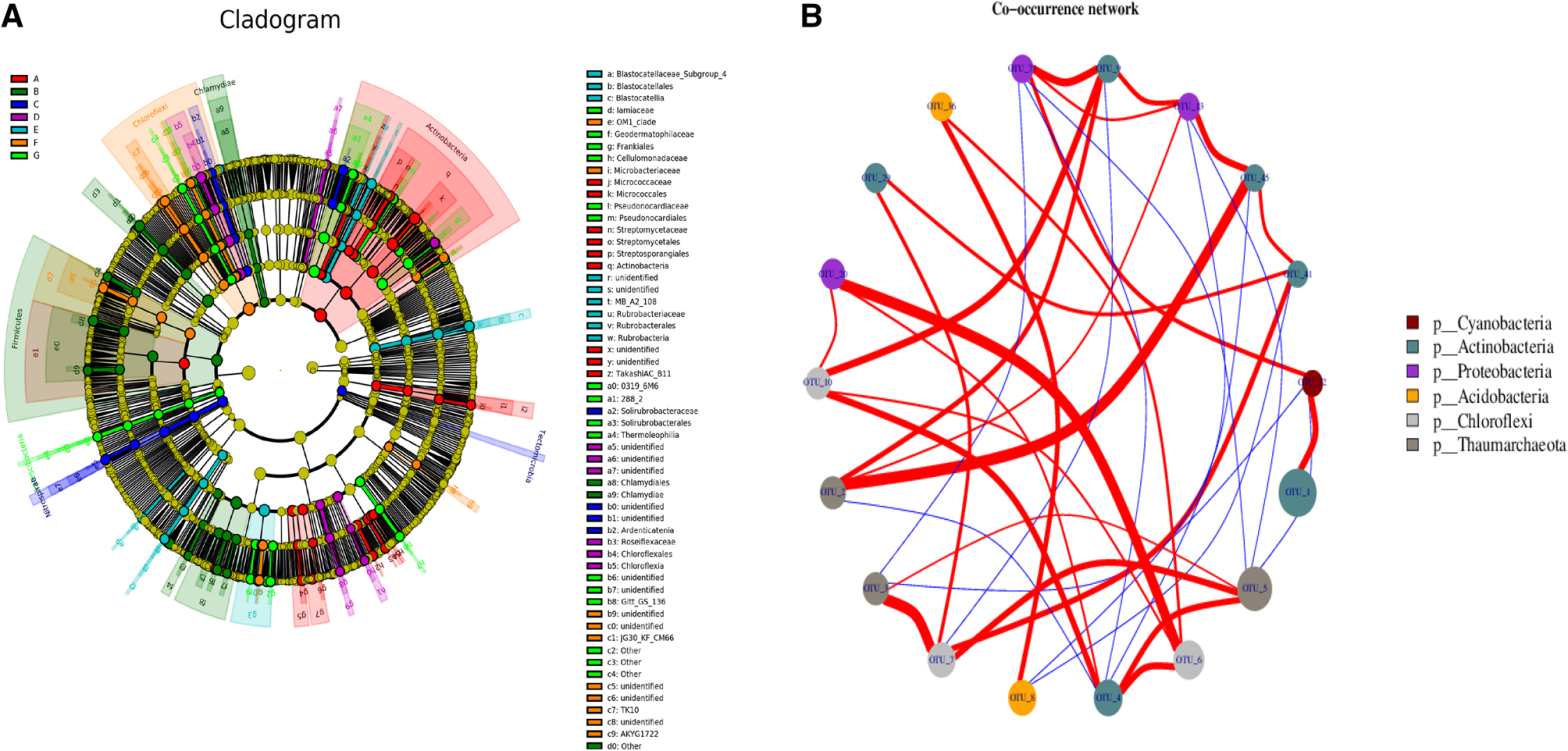
(**A**) The cladogram of soil rhizospheric bacteria generated by LEfSe indicating differences of taxa between treatments. The connection stands for a strong and significant occurelation. The size of each node is proportional to the relative abundance. Red, dark green, blue, purple, grass green, orange, and light green on the bacterial trees indicate the taxa that are enriched in cropping year of 5, 10, 15, 20, 25, 30 and 30 with field corn, respectively (ɑ > 0.01). (**B**) The co-occurrence network interactions of soil rhizospheric bacteria. The connection stands for a strong and significant ocrrelation. The size of each node is proportional to the relative abundance.

The relative abundance of the assigned bacteria phyla and genera was compared among the different samples (Fig. 5). The results showed that Proteobacteria, Actinobacteria, Chloroflexi, Planctomycetes, and Acidobacteria were located in the top five among all bacteria phyla, which counted 22.46–26.71%, 15.70–24.43%, 11.6–15.45%, 8.65–10.91%, and 8.07–10.38% of different cropping year, respectively. Proteobacteria was enriched in rhizosphere soils of 10 years cropping, whereas, Gemmatimonadetes and Thaumarchaeota were depleted at 10 years cropping. Proteobacteria is a key phylum in the rhizosphere soil of continuous maize seed production (Fig. 5A). Two genera of *Flavobacterrium* and *Leptolyngbya* exhibited lower abundance, while *Pseudarthrobacter, RB41*, and *Roseiflexus* exhibited higher abundance in different soil samples, and the relative abundance of which accounted for 0.02–0.72%, 0–1.32%, 0.97–5.75%, 0.77–2.43%, and 0.71–2.23%, respectively. *Haliangium and Streptomyces* were also presented in the soils, which occupied 0.84–1.4% and 0.38–0.86% in different cropping year (Fig. 5B). Long-term mono maize seed cropping enriched the amount of *Roseiflexus* and *Blastococcus* in maize rhizosphere soil. Continuous cropping induced large changes in the bacterial flora in maize rhizosphere soil. According to analysis of Lefse, Actinobacteria and Cyanobacteria were dominated at 5–10 years cropping, while, *Chloroflexi* and *Roseiflexus* were over-presented at 20 years cropping. *Blastocatellaceae-subgroup-4* and *Rhodocyclaceae* was dominant at 25 years cropping, *Thermoleophilia* and Solirubrobacterales were dominated at 30 years cropping with field maize, while *Chloroflexi* and *Bacilli* was dominated at 30 years cropping without field maize (Fig. 5C).To gain deeper insight into the differences between the groups, a profile clustering network analysis was applied. The profile obtained by a Cytoscape network analysis showed all the phyla and highlighted the distribution and relative abundances of all phyla of different cropping years(Fig. 6A,B). Five years cropping enriched Actinobacteria, 10 years cropping has relative abundance of Firmicutes, 15 years cropping highlighted Nitrospirae and Tectomicrobia, and 30 years cropping highlighted Chloroflexi. From family level, 10 years cropping enriched Geodermatophilaceae and OM1_clade, 15 years cropping enriched Blastocatellia and OM1_clade. Thirty years cropping with field maize enriched Blastocatellaceae_sugbgoup_4, Geodermatophilaceae, Blastocatellia, OM1_clade, Geodermatophilaceae, Frankiales, Pseudonocardiaceae and Cellulomatophilaceae, while 30 years cropping without field maize enriched Blastocatellia, OM1_clade, Frankiales and Lamiaceae. Significant difference of relative abundance on different cropping year of different phyla and genera were clearly observed (*P* < 0.01). OTU_20 of Proteobacteria was more correlated to OTU_6 of Chloroflexi, and OTU_2 of Thaumarchaeota was more correlated to OTU-45 of Actinobacteria. Co-occurrence of different phyla was clearly showed as well (Fig. 6A,B).

### Relationship between disease incidence and microbial community composition

**Figure 7:**
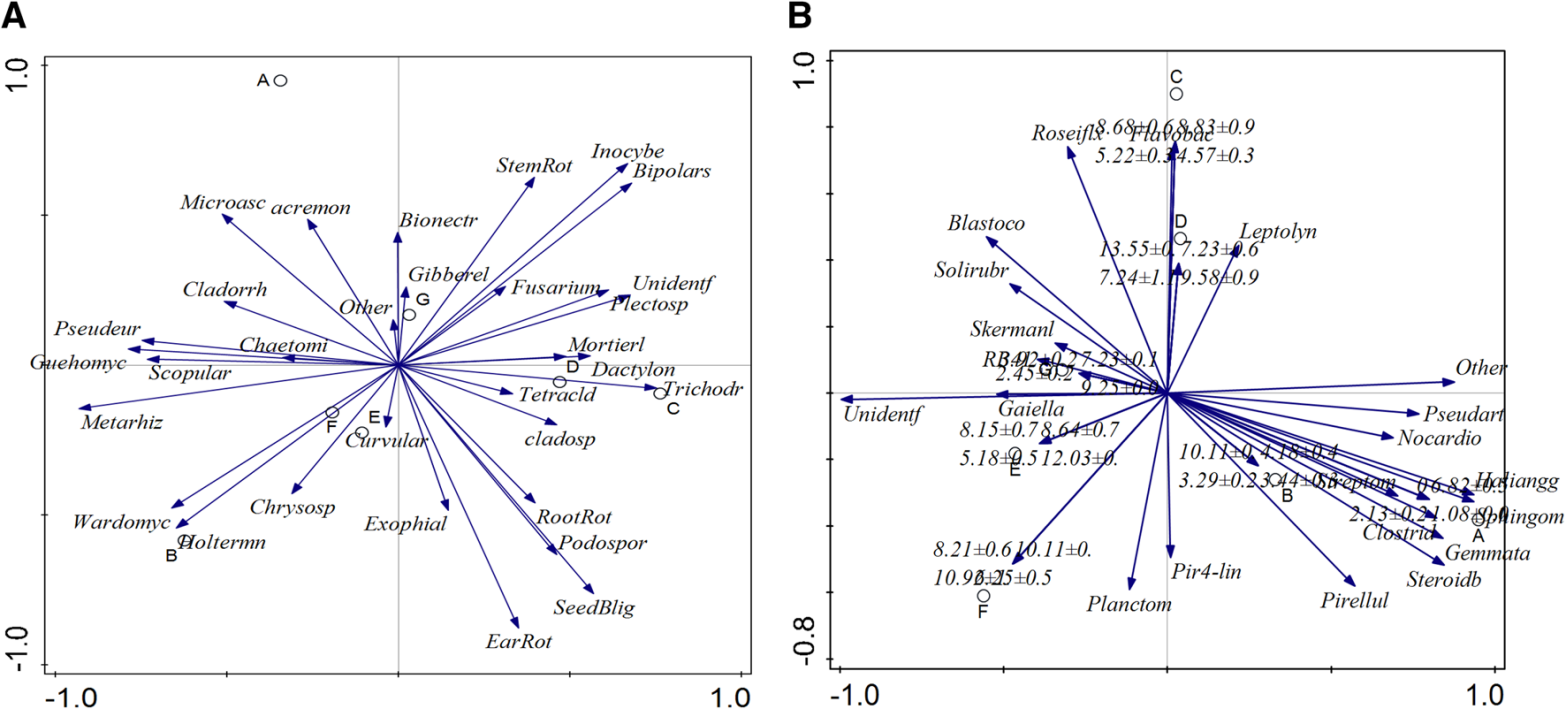
(**A**) RDA analysis of the relationship between the disease incidence and relative abundance of fungal genus of different cropping years. Correlation between different disease incidence and RDA axes were represent by the length and angel of arrows. (**B**) RDA analysis of the relationship between the disease incidence of different continuous cropping year and relative abundance of bacteria genus of different cropping years. Correlation between different disease incidence and RDA axes were represent by the length and angel of arrows.

From the results of RDA of fungi, root rot, seedling blight, and ear rot showed the similarly variation trends, and their disease incidence was significant positively related with *Podospora, Trichoderma, Tetracladium* and *Exophiala*. While, above mentioned diseases were significant negatively related to *Microascus, Cladosporium, Acremonium, Chaetomium,* and *Pseudeurotium*. Stem rot was significant positively related to *Fusarium, Gibberella, Bipolaris, Plectosphaerella*, while the disease incidence of it was significant negatively correlated to *Wardomyces, Chrysosporium, Curvularia* and *Holtermanniella*. Different pathogenic pathogen led to different disease incidence in different continuous cropping year (Fig. 7A).

From the results of RDA of bacteria, the disease incidence of continuous cropping of 5 years was significant positively correlated to *Pseudarthrobacter, Gemmata, Steroidobacter, Pirellula, Sphingomonas, Halianggium, Nocardioides, Clostridiu, Pir4-lineage, Pirellula, Streptomyces,* and 10 years cropping was positively related to all above mentioned bacteria. Continuous cropping of 15 years was significant positively related to *Flavobacterrium* and *Leptolyngbya*, and 20 years cropping was positive related to *Pseudarthrobacte*. Cropping 30 years was positively related *Planctomyces,Flavobacterrium*, and genus U*nidentified*, and cropping 30 years was significant positively related with them. Continuous cropping of 25 years and 30 years with field maize was significant positively related to genera *Roseiflexus, Blastococcus, Solirubrobacter, RB41,* and *Skermanella* (Fig. 7B).

## Discussion

Study results showed that soil microbial communities are influenced by multiple factors such as plant type, soil properties^16^, and fertilization^17^. Long-term fertilization is correlated with differences in both composition and community-level physiological profile of soil microbial communities^18^. Inappropriate fertilization can reduce bacterial diversity^19^. Present study is a long-term chemical fertilization system and located in an arid and semi-arid region. Ascomycota, Basidiomycota and Mortierellomycota were dominant phyla after long-term continuous cropping of maize seed production, which was consistent with the results of previous study^7^. Ascomycota tended to reside in cooler and arid area because of their evolutionary histories^20^, and its many species play a major ecological role as decomposers^21^. Our research was also consistent with Lauber et al^22^, who set forth that higher Ascomycota than Basidiomycota in P-rich soils (8.96–12.51 mg/kg). At the genus level of fungi, *Mortierella* and *Chrysosporium* dominated in rhizosphere soil, *Fusarium*, *Giberrella*, and *Pseudeurotium* were the important microbe for community composition. The most abundant genera in our study play important roles in different continuous cropping year because the highest abundance of sequences belonged to different species. The abundance of most dominated *Mortierellales* decreased with continuous cropping year indicating a decrease of protective microbe^7^. Proteobacteria comprised of 22.46–26.71% of all bacterial communities in different continuous cropping year, confirming that it is still an important soil bacterial group after 30 years monoculture. Specific taxa of Actinobacteria, Acidobacteria, and Proteobacteria showed significant shifts across the increased cropping year. The relative abundance of Proteobacteria and Actinobacteria decreased, while that of Acidobacteria increased with the continuous cropping, which was consistent with Lauber et al.^23^,who pointed out that Proteobacteria, Actinobacteria and Acidobacteria were the top three phyla in the soil. At the genus level, as many of the less abundant groups of Actinobacteria showed a propensity for soil environment with reduced carbon or nutrient availability. *Chloroflexi* occupied a certain property in our studied area, though it is un-culturable^23^. Results showed that Proteobacteria and Actinobacteria were greatly responsive to soil pH and SOC^24^ and the decrease of Proteobacteria and Actinobacteria may be caused by the decrease of soil organic matter and pH value^1^. Difference of microbial community may be caused by the difference of the physicochemical properties, such as soil type or pH under the same management^25,26^ and land use type^24^. The possible explanation for the different variation of microbial community of field G with the other fields might related to the effects of its field management which was mostly coherent with its edaphic matrix. Seed maize’s pollination, production model, parents, yields and residues treatment are greatly differed with that of field maize. Field maize has higher yield, loosely pollination, stubble returning, and lower dose fertilization. Influence on the field G from the field management were obviously visible from planting history.

Intensive research attempts are underway to improve plant growth and tolerance to various abiotic stresses, and to protect plants from soil borne pathogens using plant growth promoting bacteria^27–30^. While, optimum environment conditions are needed for growth of some fungal colonies, because they failed to synthesize some key compounds by lacking of some key conditions, and fungi are widely different on obtaining nutrients from the medium of basal. Some biocontrol genera presented in rhizosphere soil of maize such as *Halianggium* and *Streptomyces*, while the abundance of the genus decreased with the increase of continuous cropping year. *Haliangium* can produce haliangicin that can inhibit the growth of a wide spectrum of fungi^31^. *Streptomyces* usually inhabit soil and can produce tubercidin and phosphlactomycin that was effective to biological control^32^. *Podospora* and *Chaetomium* can potentially decrease the population of harmful fungi and act as biological control to pathogen in the field, and *Cladorrhinum* has the beneficial effect on increasing absorption of phosphorus in the field. Similarly, rhizosphere *Pseudomonad* produced antibiotics, and rhizosphere *Bacilli* produced lipopeptides and polyketides, biosurfactants, and volatile organic chemicals^33–36^. It is worthy that the relative decrease of benificial microbe of *Podospora, Chaetomium,* and *Cladorrhinum* will potentially lead to decrease of beneficial secretions around rhizosphere. Our results confirmed that long term mono cropping of maize seed production caused a decrease of biocontrol genera and a decline of plant beneficial microbe in the rhizosphere soil.

Rhizosphere fungal communities are considered critically important for plant health and soil fertility. Generally, continuous cropping practices and severe root-rot disease affected the community structure and diversity of rhizosphere and root endophytic fungi^37^. Root associated microbes, including endophytes, closely cooperated with each other and can mediate important physiological processes, especially nutrient acquisition and plant fitness to abiotic stresses^38,39^. In the present study, most of those relative increased genera as *Fusarium, Gibberella, Bipolaris, Acremonium, Plectosphaerellaceae, Curvularia,* and *Cladosporium* are potential phytopathogens caused maize disease such as root rot, stem root, and ear rot in continuous cropping. With seriously harm to the agroecosystem, the eradication of above mentioned pathogens is difficult with the pathogens accumulating in cultivated soils and then significantly impact maize seed production. *Fusarium* was considered the major pathogen of root rot^40^, and *Gibberella* was considered the major pathogen of ear rot. *F. oxysporum* f. sp. *Niveum* (FON) is considered to be the most important soil-borne facultative pathogen, causing economically important losses and limiting production in many areas of the world^11^. Genus *Bipolaris* includes important plant pathogens with worldwide distribution^41^, and *Curvularia* leaf spot, caused by *Curvularia lunata* (Wakker) significantly reduces the yields of maize^42^. As evidence from the disease incidence of 4 different disease, the increase of *Fusarium*, *Gibberella, Bipolaris, cladosporium,* and *Acremonium* increased relative abundance of potential pathogen in different cropping year, and the decreased diversity of *Podospora, Chaetomium,* and *Cladorrhinum* suggesting that the plant protection decreased and the maize is in danger of pathogen invasion. More attention should be devoted to make clear the variation of those microbial communities such as *Fusarium*, *Gibberella* and *Bipolaris* etc*.* Study results showed that an optimum microbial community can promote soil defense capability^43^. It has been demonstrated that some root associated microbes may reduce the peroxidation of membrane lipids, thereby strengthening the ability of membranes to withstand abiotic stress^39,44^.

Disease severity is the intensity of disease prevalence based on specific scale of rating as described in the results of Lambert and Whites^45^. The spores of the pathogen can survive in the soil for several years which increased the difficult to disease control or prevention. Once the pathogen is established in soil, the soil will be unsustainable to maintain the plant production. In the present study, different continuous cropping fields under the same management, same climate conditions and similar soil properties indicated different disease incidence and severity led to different variation of soil microbial community. In many instances, these diseases are caused by a complex of fungal species^7^. In the same way, pathogenic pathogen caused four diseases in the study are a complex of fungal species, therefore, different pathogenic pathogen dominated at different cropping year led to different disease and disease incidence. Furthermore,variations in rhizosphere microbial community structure may also make the root more susceptible to pathogen infection and appeared different disease incidence. In the studied area, disease severity increased of maize root-rot and stem-rot in July and August, as environmental and weather conditions are optimal for the pathogens invasion. Novel and adaptive methods should be developed to disease control and plant production. PCoA results showed that the samples of the same land use types were often clustered but 30 years cropping with field maize were independent with the other years cropping. RDA analysis also confirmed a closely correlation of plant–microbe between the different disease incidence and microbial community structure and diversity in our study. Our results indicated that optimizing the soil microbial community under factors matric to an appropriate level was urgently needed to keep the plantation healthy.

## Conclusions

Continuous cropping of maize seed production increased disease incidence with the increase of cropping year, while the highest disease incidence and disease severity of four diseases varied among different cropping years. Mono-maize cropping led to significant difference in the microbial community and diversity among different continuous cropping year. Fungal community diversity increased with the increase of continuous cropping year with exception of 10 years. Ascomycota, Mortierellomycota and Basidiomycota dominated top 3 fungal flora. A certain proportion of pathogenic *Fusarium, Gibberella, Cladosporium* etc. were detected in the rhizosphere fungi in different continuous cropping year. The relative increase of *Fusarium, Gibberella, Cladorrhinum*, and the relative decrease of *Mortierella, Chrysosporium, Podospora, Chaetomium,* and *Exophiala* with the increase of continuous cropping year potentially contributed to the structural segregation of different cropping year. Proteobacteria, Actinobacteria and Chloroflexi were located in the top 3 among all bacteria phyla. *Haliangium and Streptomyces* were also found in the soils with a significant decrease with the increase of cropping year. Continuous maize seed production led to the decrease of protective and biocontrol genera, and increased the potential pathogenic pathogen. While relative abundance of potential pathogen varied among different cropping year and led to different disease and incidence. There was a closely correlation between disease incidence and variation of microbial community. Pathogenic disease incidence increased with the increase of cropping year, and the cropping plant is in danger of pathogen invasion. Optimizing the soil microbial community under factors matric to an appropriate level was urgently needed to keep the maize seed production healthy.

## Material and methods

### Site description

The study site were located at Shajing town of Zhangye, Gansu province, China, where more than 30 companies with more than hundreds of maize varieties and nearly 3,000 hectares fields were operating for many years. The soil was named as irrigated desert soil with organic matter of 18.56–20.34 g kg^−1^, available-nitrogen of 45–52 mg kg^−1^, available-phosphorus of 8.96–12.51 mg kg^−1^, available-potassium of 164–168 mg kg^−1^, and pH of 8.33–8.45. The annual temperature, evaporation, and precipitation were 4 °C, 2000–2100 mm, and 200–243 mm, respectively. Seven site with continuous mono-cropping year of 5 years (A, 39°42′52″N, 100°15′30″E), 10 years (B, 39°42′52″N, 100°15′30″E ), 15 years (C, 39°5′14″N, 100°15′42″E), 20 years (D, 39°5′46″N, 100°16′29″E), 25 years (E, 39°5′17″N, 100°15′40″E), 30 years (F, 38°59′46″N, 100°20′20″E) and 30 years (G, 38°58′35″N, 100°19′53″E) were selected for our study. Field G has 3 years field maize planting history (Conventional grown, no isolation, the straw is crushed and returned to the field) and all the other fields were under the same fertilization and management mode, and the area of each plot was 36m^2^ (4 × 9) and was separated with the others by isolation.

### Soil sample collection

The soil samples of rhizosphere of different mono cropping year were collected after harvest with shaking the soil particles adhering to the maize roots. Each sample was a mixture of rhizosphere soil from 10 plants, and then the root hair, stone and plastic pieces were picked out by hand. Three duplicated samples of the mixture about 5 g was put into the ice box and transferred to the laboratory at 1 h for further testing. The samples for high throughput sequence test were preserved at − 20 °C before posting and posted with dry-ice.

### Disease incidence statistics

Incidences of seedling blight, root rot and stem rot were investigated at the seedling stage and the flare opening stage on the basal stem, respectively, while incidence of ear rot was investigated at seedling and adult-plant stage. The diseased plant was washed with running tap water, and assessed for the presence and severity of root rot, stem rot, seedling blight, and ear rot symptoms on a 0–4 scale according to Zhou et al^46^, where: 0 means no symptom, 1 means mild symptom, 2 means obvious lesions, 3 means severe lesions on the stem and diminished plant vigor. Disease incidence (%) = number of infected plants × 100/ Total number of plants in each block.

DNA extraction, PCR amplification and illumina Miseq sequencing of the soil.

The DNA of samples was extracted by using the MOBIO PowerSoil DNA Isolation Kit^47^. The genomic DNA was amplified by PCR using the 16S rRNA gene V4 region primers (515a–806a). The primers sequence were: GTGCCAGCMGCCGCGGTAA, and GGACTACHVGGGTWTCTAAT. PCR conditions: Denaturation for 5 min at 94 °C, followed by 26 cycles of 30 s at 94 °C, 30 s at 50 °C, and 60 s at 72 °C. All reactions ended with a final extension of 7 min at 72 °C. The amplified product was verified by 1% agarose gel electrophoresis. The PCR amplification product was further purified by using a purification kit, and the PCR mixed product was recovered by using an EZNA Gel extraction kit (Omega, USA). The illumina Misequence PE 250 (Beijing Allwegene Biotechnology Co. LTD., China) was used for sequencing and microbial community analysis.

### Statistical analysis

The sequence data were processed on the quantitative insights into Microbial Ecology platform according to Caporaso et al^48^. The 97% similarity of sequence was used as a criterion of the classification of the Operational Taxonomic Units (OTU). The Chao 1 and Shannon diversity were calculated by using the QIIME platform (http://qiime.org/). The open source software Cytoscape 2.8^49^ was employed to visualize all the phyla identified based on total read counts. Redundancy analysis (RDA) of the relationship between microbial biomass composition and the disease incidence was carried out by using the CANOCO 5 software. A metagenomic biomarker discovery approach was employed with LEfSe (Linear discriminant analysis) coupled with effect size measurement which performs a nonparametric wilcoxon sum-rank test to assess the effect size of each phylum^50^. The disease incidence were statistically determined with Microsoft Excel and Duncan’s multiple range test was applied when the one-way ANOVA showed obvious differences (*p* < *0.05*).
